# Supplementary material for: Evolutionary selection of biofilm-mediated extended phenotypes in Yersinia pestis in response to a fluctuating environment
Source: Nat Commun. 2020 Jan 15;11:281. doi: 10.1038/s41467-019-14099-w (PMC6962365; doi:10.1038/s41467-019-14099-w)
Supplement: Supplementary file 1 — Supplementary information [file 41467_2019_14099_MOESM1_ESM.pdf]

Supplementary Information

Evolutionary selection of biofilm-mediated extended phenotypes in *Yersinia pestis* in response to a changing environment

Cui, Schmid et al.

Content

Supplementary Figures 2
  Supplementary Figure 1 2
  Supplementary Figure 2 4
  Supplementary Figure 3 5
  Supplementary Figure 4 6
  Supplementary Figure 5 7
Supplementary Table 8
  Supplementary Table 1. 8
Supplementary References 9

Supplementary Figures

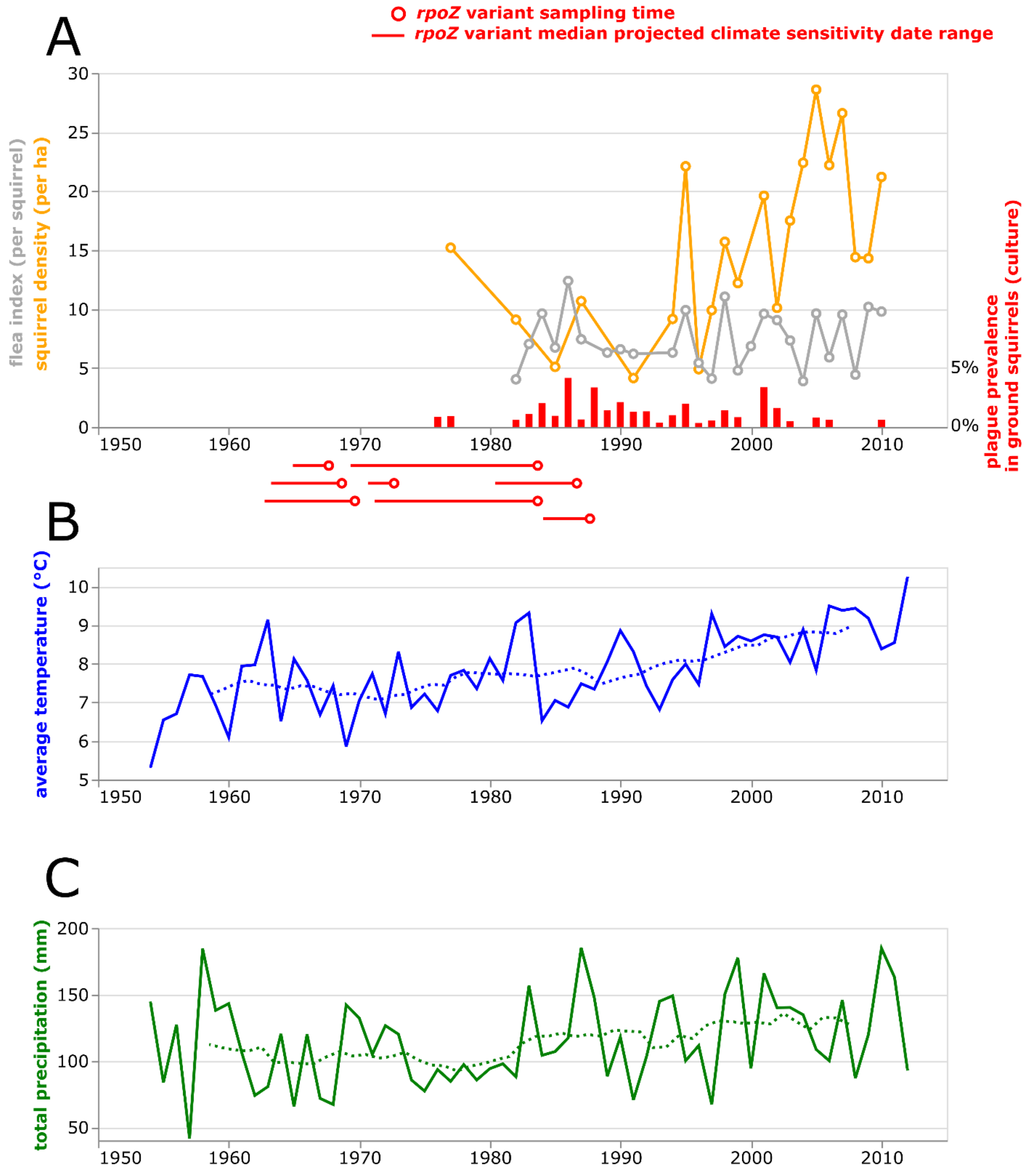

Supplementary Figure 1. Summary of plague surveillance and climate information. Panel A, Surveillance data of the estimated number of ground squirrels per hectare (orange dots) and the average number of fleas per ground squirrels (i.e., flea index, grey dots) in the Guertu plague focus, as well as the prevalence of plague in the long-tailed ground squirrels measured by

culturing blood samples (red bars). Also shown are the years in which *rpoZ* variants were sampled (red dots), and the median date range in which climate might have affected the selection of these variants (red lines), based on the date of divergence of the *rpoZ* variant-containing branches of the phylogeny of plague in Guertu. Surveillance data does not go as far back as the stored *Y. pestis* samples, and only directly overlaps the *rpoZ*-variant years in 1983 and 1987 for the flea index, and in 1987 for the ground squirrel density per hectare. Panel B&C, Average annual temperature and total annual precipitation recorded from the weather station nearest to the Guertu ecosystem (station 51334, solid lines) and the 10-year moving average (dotted lines). From the 70's onwards, both temperature and precipitation have been rising in the Guertu ecosystem.

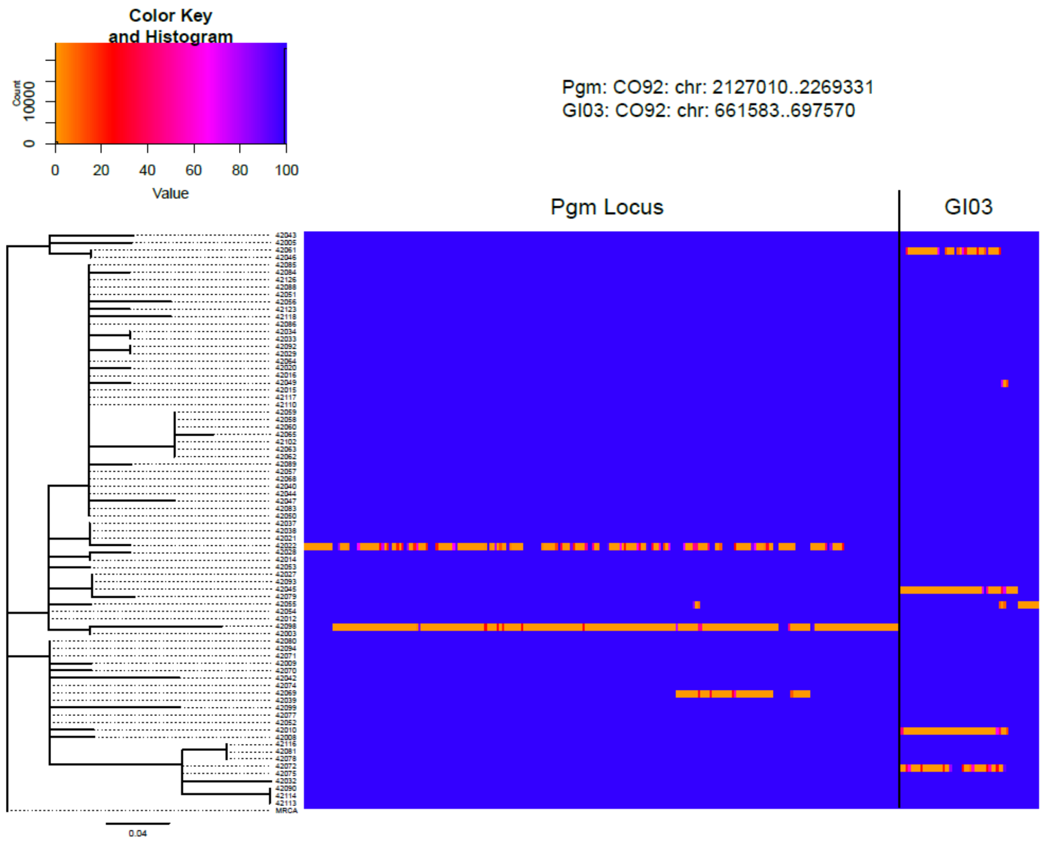

Supplementary Figure 2. Genome fragment loss of Guertu genomes. The left is the MLtree of 78 Guertu genomes based on SNPs. Each row in the heat map indicates one genome that corresponds with the tree, and each column indicates a 500 bp length fragment from the broken accessory genome. All fragments to the left of the black line are located on the *pgm* locus, and the fragments to the right are on the genome island GI03.

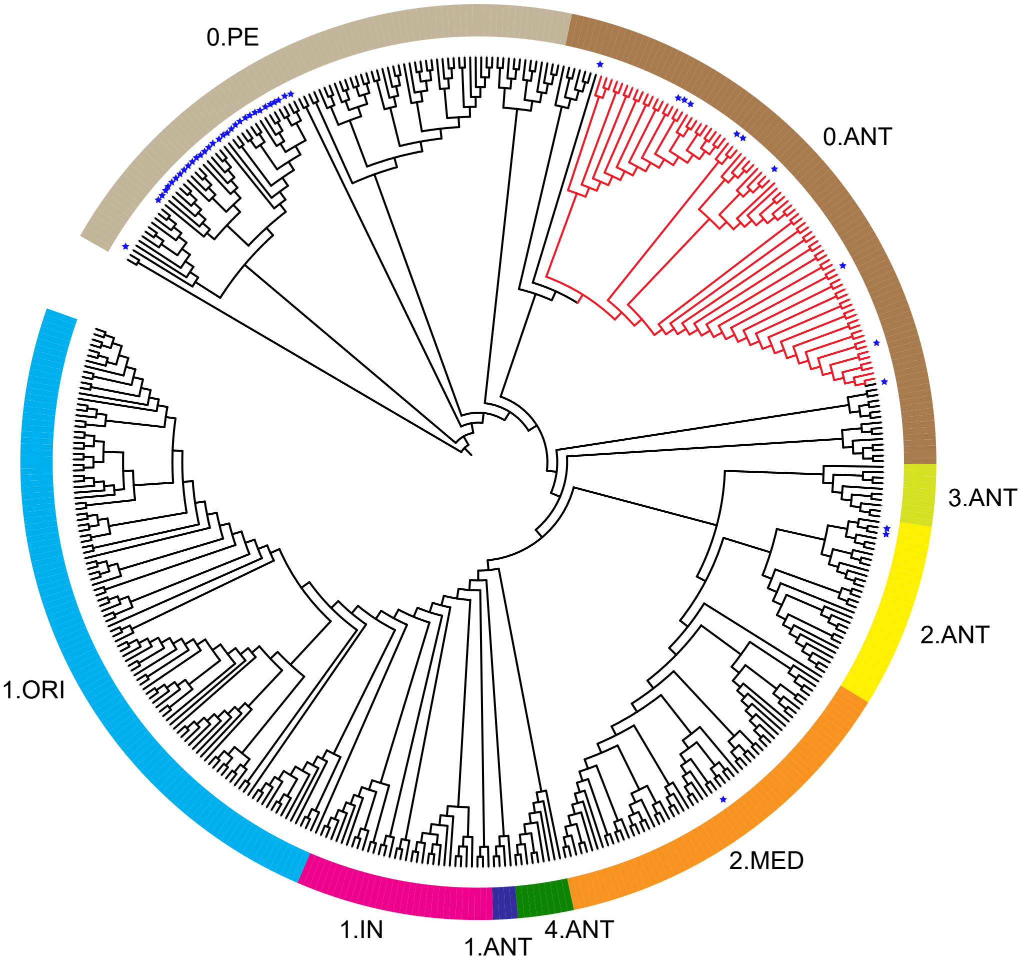

Supplementary Figure 3. Maximum likelihood tree of 78 Guertu genomes and previously sequenced 368 *Y. pestis* genomes based on SNPs. The plot was generated using iTOL, the branch length information was omitted and topology is only indicated for visual effect. The red branch indicates Guertu isolates and the stars are strains that carried mutation in the *rpoZ* gene. The color band surrounding the tree indicates the phylogroups of *Y. pestis*.

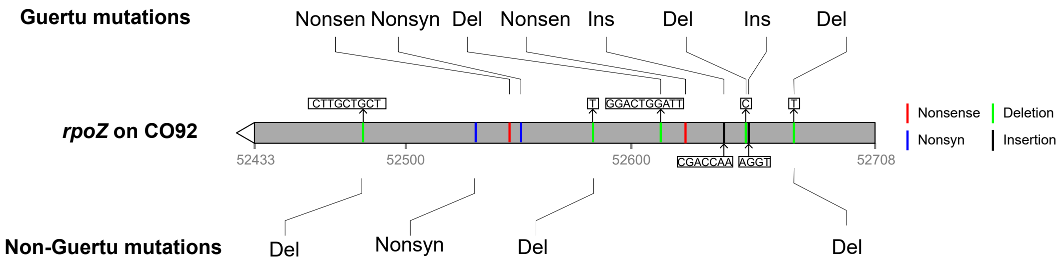

Supplementary Figure 4. The mutations observed in *rpoZ* gene of *Y. pestis*. In total 11 different types of mutations were observed in *Y. pestis*. The color bars indicated the position of mutations in the CO92 genome.

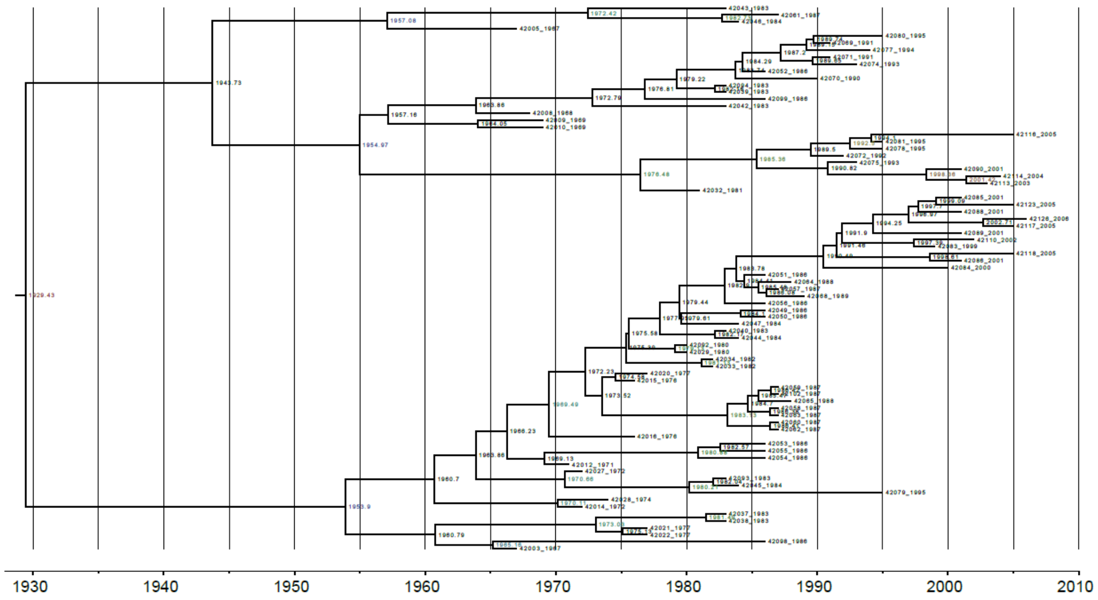

Supplementary Figure 5. Maximum clade credibility tree of the 78 Guertu isolates. The tree is scaled in AD years, and the number on each node indicates the possible year of branch splitting.

Supplementary Table

Supplementary Table 1. Differences in SNPs or InDels between the *rpoZ* variants (orange) and the two *rpoZ* references (grey) in the biofilm formation experiment.

**Isolate ID:**			42003	42042	42009	42027	42008	42056	42057	42043	**42080**	**42054**		
Relative biofilm formation (mean of 6 repeats):			**557**	**273**	**198**	**172**	**124**	**89**	**55**	**55**	**38**	**8**		
**Mutation ID(s)**	**GENE (name)**	**Mutation type**											***Yersinia pestis* CO92 (NC003143) description[#1]**	**Suggested function from Literature**
**SNP001-3, ID0001-5**	YPO0039 (rpoZ)	NS (1x), nonsense (2x), del (5x)	**X**	**X**	**X**	**X**	**X**	**X**	**X**	**X**			•**DNA-directed RNA polymerase subunit omega**: promotes RNA polymerase assembly or stability; latches the N- and C-terminal regions of the beta subunit thereby facilitating its interaction with the beta and alpha subunits	Biofilm (this paper)
**SNP007**	YPO0348 (aspA)	NS	**X**							**X**			•**aspartate ammonia-lyase**: catalyzes the formation of fumarate from aspartate	Virulence [1]
**ID0012**	YPO0377	del	**X**	**X**	**X**	**X**	**X**	**X**	**X**		**X**	**X**	•**hypothetical protein**: similar to several proteins of unknown function including: *Escherichia coli* hypothetical 7.2 kDa protein in hflC-purA intergenic region YjeT	Biofilm [2]
**ID0016**	Intergenic, between YPO0435 and YPO0436 (deoC)	ins		**X**	**X**		**X**				**X**		•**Na+ dependent nucleoside transporter family protein**: similar to *Vibrio cholerae* NupC family protein Vc2352, and to *Helicobacter pylori* J99 nucleoside transporter Jhp1106 •**deoxyribose-phosphate aldolase**: catalyzes the formation of D-glyceraldehyde 3-phosphate and acetaldehyde from 2-deoxy-D-ribose-5-phosphate	Starvation [3]
**SNP009, ID0017**	Intergenic, YPO0535 (leuO) - YPO0536	Intergenic, del						**X**	**X**				•**leucine transcriptional activator:** activator for leuABCD operon; member of LysR family of transcriptional activators •**hypothetical protein**: doubtful CDS with no significant database hits	Cold temperature [4]
**SNP013**	YPO0776	NS	**X**			**X**		**X**	**X**			**X**	•**siderophore biosysnthesis protein:** similar to N-terminus of *Yersinia pestis* yersiniabactin biogenesis protein Irp2, and to *Pseudomonas aeruginosa* pyochelin synthetase PchF	Virulence [5]
**ID0019**	YPO0782	ins			**X**						**X**		•**hypothetical protein**: poor database matches. Doubtful CDS	-
**SNP017**	YPO1029 (gcvA)	NS					**X**						•**DNA-binding transcriptional activator GcvA**: glycine cleavage system transcriptional activator; activates the gcvTHP operon in the presence of glycine and represses the operon in its absence	Biofilm [6]
**ID0022**	Intergenic, between YPO1074 and YPO1075 (dkgB)	ins								**X**			•**D,D-heptose 1,7-bisphosphate phosphatase:** converts the D-glycero-beta-D-manno-heptose 1,7-bisphosphate intermediate into D-glycero-beta-D-manno-heptose 1-phosphate •**2,5-diketo-D-gluconate reductase B**: catalyzes the reduction of 2,5-diketo-D-gluconic acid to 2-keto-L-gulonic acid	Biofilm [6]
**SNP018**	YPO1108 (gltA)	NS						**X**					•**type II citrate synthase type II enzyme**: in *Escherichia coli* this enzyme forms a trimer of dimers which is allosterically inhibited by NADH and competitively inhibited by alpha-ketoglutarate; allosteric inhibition is lost when Cys206 is chemically modified which also affects hexa	Quorum Sensing [7]
**ID0023**	YPO1190	del		**X**									•**ABC transport ATP-binding subunit**: similar to *Bacillus halodurans* ABC transporter BH0248, and to *Escherichia coli* high-affinity branched-chain amino acid transport ATP-binding protein LivG	ABC Transporter [8]
**ID0027**	YPO1522 (metG)	ins	**X**	**X**	**X**	**X**	**X**	**X**		**X**	**X**	**X**	•**methionyl-tRNA synthetase methionine--tRNA ligase**: MetRS; adds methionine to tRNA(Met) with cleavage of ATP to AMP and diphosphate; some MetRS enzymes form dimers depending on a C-terminal domain that is also found in other proteins such as Trbp111 in *Aquifex aeolicus* and the cold-shock protein CsaA from *Bacillus subtilis* while others do not; four subfamilies exist based on sequence motifs and zinc content	Protein biosynthesis [9]

**SNP026**	YPO1650	NS								X			•**hypothetical protein**: Weakly similar to *Mycobacterium tuberculosis* phosphoribosylamine--glycine ligase PurD or RV0772 or MTCY	-
**ID0029**	Intergenic, Between YPO1657 and YPO1659	del		X	X		X				X		•**methyl-accepting chemotaxis protein (pseudogene)**: Similar to *Escherichia coli* methyl-accepting chemotaxis protein III Trg or B1421<br>•**hypothetical protein**: no significant database hits	-
**ID0031**	YPO1704 (proQ)	ins							X				•**solute/DNA competence effector:** affects solute and DNA transport through an unknown mechanism	Biofilm [10]
**ID0034-35**	YPO2048 (cutC)	ins, del	X		X								•**copper homeostasis protein CutC:** similar to *Escherichia coli* copper homeostasis protein CutC, and to *Homo sapiens* hypothetical protein CGI-32	Copper homeostasis / virulence [11]
**ID0039**	Intergenic, YPO2308 (rstA) - YPO2309 (rstB)	del								X			•**DNA-binding transcriptional regulator RstA**: response regulator in two-component regulatory system with RstB<br>•**sensor protein RstB**: similar to *Escherichia coli* sensor protein RstB, and to *Escherichia coli* sensor protein CpxA	Stress survival [12]
**ID0040**	YPO2318 (asr)	ins			X			X	X				•**acid-shock protein**	Acid shock survival
**ID0042**	YPO2401	del					X						•**cysteine desulfurase:** with SufBC activates cysteine desulfurase SufS	Iron starvation [13]
**SNP040**	Intergenic, YPO2449 - YPO2450 (tqsA)	SNP								X			•**LuxR family transcriptional regulator**: similar to *Salmonella typhi* colanic acid capsular biosynthesis activation protein A RcsA, and to *Escherichia coli* colanic acid capsular biosynthesis activation protein A RcsA S	Biofilm [14,15]
**ID0056**	YPO2872 (xseA)	del	X	X	X	X		X	X	X	X	X	•**exodeoxyribonuclease VII**: large subunit bidirectionally degrades single-stranded DNA into large acid-insoluble oligonucleotides	DNA degradation [16]
**ID0057**	YPO2892 (hscA)	del									X		•**chaperone protein HscA**: involved in the maturation of iron-sulfur cluster-containing proteins	Iron metabolism [17]
**ID0059**	YPO3094 (rosB)	del						X					•**cation:proton antiport protein YbaL**: member of the CPA-2 family of antiporters; uncharacterized protein	Innate immune sys defense [18]
**ID0068**	YPO3404	del			X		X				X		•**hypothetical protein**: similar to *Escherichia coli* hypothetical protein YadE. NodB-like according to pfam (http://pfam.xfam.org/family/PF01522.20)	Biofilm [18]
**ID0069**	Intergenic, YPO3553 (elbB) - YPO3555 (arcB)	del										X	•**hypothetical protein:** no significant database hits<br>•**aerobic respiration control sensor protein ArcB**: sensor-regulator protein which regulates the expression of many genes in response to respiratory growth conditions including anaerobic repression of the arc modulon; hybrid sensory histidine kinase in two-component regulatory system with ArcA, regulates respiration and fermentation, senses oxidized quinones	Biofilm [18]

#1 http://cloud.im.ac.cn/dataservice/search_entry.shtml?db=bacteria_genome&id=NC_003143&type=1

Supplementary References

1 Bearden, S. W. *et al.* Attenuated enzootic (pestoides) isolates of *Yersinia pestis* express active aspartase. *Microbiology* **155**, 198-209 (2009).

2 Yepes, A. *et al.* The biofilm formation defect of a *Bacillus subtilis* flotillin-defective mutant involves the protease FtsH. *Mol. Microbiol.* **86**, 457-471 (2012).

3 O'May, G. A. *et al.* The high-affinity phosphate transporter Pst in *Proteus mirabilis* HI4320 and its importance in biofilm formation. *Microbiology* **155**, 1523-1535 (2009).

4 Klauck, E., Böhringer, J. & Hengge-Aronis, R. The LysR-like regulator LeuO in *Escherichia coli* is involved in the translational regulation of *rpoS* by affecting the expression of the small regulatory DsrA-RNA. *Mol. Microbiol.* **25**, 559-569 (1997).
5 Perry, R. D. & Fetherston, J. D. Yersiniabactin iron uptake: mechanisms and role in *Yersinia pestis* pathogenesis. *Microbes Infect.* **13**, 808-817 (2011).
6 Niba, E. T. E., Naka, Y., Nagase, M., Mori, H. & Kitakawa, M. A genome-wide approach to identify the genes involved in biofilm formation in *E. coli*. *DNA Res.* **14**, 237-246 (2007).
7 Yu, J. *et al.* Analysis of Autoinducer-2 Quorum Sensing in *Yersinia pestis*. *Infect. Immun.* **81**, 4053-4062 (2013).
8 Schneider, E. & Hunke, S. ATP-binding-cassette (ABC) transport systems: functional and structural aspects of the ATP-hydrolyzing subunits/domains. *FEMS Microbiol. Rev.* **22**, 1-20 (1998).
9 Lemoine, F., Waller, J. P. & van Rapenbusch, R. Studies on methionyl transfer RNA synthetase. 1. Purification and some properties of methionyl transfer RNA synthetase from *Escherichia coli* K-12. *Eur J Biochem* **4**, 213-221 (1968).
10 Sheidy, D. T. & Zielke, R. A. Analysis and expansion of the role of the *Escherichia coli* protein *ProQ*. *PLoS One* **8**, e79656, doi:10.1371/journal.pone.0079656 (2013).
11 Ladomersky, E. & Petris, M. J. Copper tolerance and virulence in bacteria. *Metallomics* **7**, 957-964 (2015).
12 Nam, D., Choi, E., Kweon, D.-H. & Shin, D. The *RstB* sensor acts on the *PhoQ* sensor to control expression of PhoP-regulated genes. *Mol. Cells* **30**, 363-368 (2010).
13 Gao, H. *et al.* The iron-responsive Fur regulon in *Yersinia pestis*. *J Bacteriol* **190**, 3063-3075 (2008).
14 Herzberg, M., Kaye, I. K., Peti, W. & Wood, T. K. YdgG (TqsA) controls biofilm formation in *Escherichia coli* K-12 through autoinducer 2 transport. *J. Bacteriol.* **188**, 587-598 (2006).
15 Kim, S. M. *et al. LuxR* homologue *SmcR* is essential for *Vibrio vulnificus* pathogenesis and biofilm detachment, and its expression is induced by host cells. *Infect. Immun.* **81**, 3721-3730 (2013).
16 Chase, J. W., Rabin, B. A., Murphy, J. B., Stone, K. L. & Williams, K. R. *Escherichia coli* exonuclease VII. Cloning and sequencing of the gene encoding the large subunit (*xseA*). *J Biol Chem* **261**, 14929-14935 (1986).
17 Pieper, R. *et al.* Proteomic analysis of iron acquisition, metabolic and regulatory responses of *Yersinia pestis* to iron starvation. *BMC Microbiol.* **10**, 30, doi:10.1186/1471-2180-10-30 (2010).
18 Bengoechea, J. A. & Skurnik, M. Temperature-regulated efflux pump/potassium antiporter system mediates resistance to cationic antimicrobial peptides in *Yersinia*. *Mol. Microbiol.* **37**, 67-80 (2000).
