# Supplementary material for: Evolutionary selection of biofilm-mediated extended phenotypes in Yersinia pestis in response to a fluctuating environment
Source: Nat Commun. 2020 Jan 15;11:281. doi: 10.1038/s41467-019-14099-w (PMC6962365; doi:10.1038/s41467-019-14099-w)
Supplement: Supplementary file 3 — Reporting Summary [file 41467_2019_14099_MOESM3_ESM.pdf]

nature research

Corresponding author(s): Nils Chr. Stenseth

Last updated by author(s): Nov 10, 2019

Reporting Summary

Nature Research wishes to improve the reproducibility of the work that we publish. This form provides structure for consistency and transparency in reporting. For further information on Nature Research policies, see Authors & Referees and the Editorial Policy Checklist .

Statistics

For all statistical analyses, confirm that the following items are present in the figure legend, table legend, main text, or Methods section.

n/a	Confirmed	
☐	☒	The exact sample size (*n*) for each experimental group/condition, given as a discrete number and unit of measurement
☐	☒	A statement on whether measurements were taken from distinct samples or whether the same sample was measured repeatedly
☐	☒	The statistical test(s) used AND whether they are one- or two-sided<br>*Only common tests should be described solely by name; describe more complex techniques in the Methods section.*
☒	☐	A description of all covariates tested
☐	☒	A description of any assumptions or corrections, such as tests of normality and adjustment for multiple comparisons
☐	☒	A full description of the statistical parameters including central tendency (e.g. means) or other basic estimates (e.g. regression coefficient) AND variation (e.g. standard deviation) or associated estimates of uncertainty (e.g. confidence intervals)
☐	☒	For null hypothesis testing, the test statistic (e.g. *F*, *t*, *r*) with confidence intervals, effect sizes, degrees of freedom and *P* value noted<br>*Give P values as exact values whenever suitable.*
☐	☒	For Bayesian analysis, information on the choice of priors and Markov chain Monte Carlo settings
☒	☐	For hierarchical and complex designs, identification of the appropriate level for tests and full reporting of outcomes
☒	☐	Estimates of effect sizes (e.g. Cohen's *d*, Pearson's *r*), indicating how they were calculated

Our web collection on statistics for biologists contains articles on many of the points above.

Software and code

Policy information about availability of computer code

Data collection	No software was used.
Data analysis	Genome assembly: SOAPdenovo2; Variation detection: MUMmer3.0, LASTZ 1.03, BWA0.7.5a; Phylogeny reconstruction: PhyML3.1, BEAST2; Tree visualization: iTOL v4. Custom algorithms were written in Clojure, and all code, data and README instructions needed to re-run and recreate the statistical analysis and statistical figures are available at https://doi.org/10.5281/zenodo.3279956

For manuscripts utilizing custom algorithms or software that are central to the research but not yet described in published literature, software must be made available to editors/reviewers. We strongly encourage code deposition in a community repository (e.g. GitHub). See the Nature Research guidelines for submitting code & software for further information.

Data

Policy information about availability of data

All manuscripts must include a data availability statement. This statement should provide the following information, where applicable:

- Accession codes, unique identifiers, or web links for publicly available datasets
- A list of figures that have associated raw data
- A description of any restrictions on data availability

The genome sequences deciphered in this study were stored in the NCBI database, the accession numbers are listed in Supplementary Data 2. There are 368 published Y. pestis genome sequences that were downloaded from the NCBI database. The accessions of genomes used are listed in Supplementary Data 5. The climate data and surveillance data are included in Supplementary Data 1.

Field-specific reporting

Please select the one below that is the best fit for your research. If you are not sure, read the appropriate sections before making your selection.

☐ Life sciences ☐ Behavioural & social sciences ☒ Ecological, evolutionary & environmental sciences

For a reference copy of the document with all sections, see nature.com/documents/nr-reporting-summary-flat.pdf

Ecological, evolutionary & environmental sciences study design

All studies must disclose on these points even when the disclosure is negative.

Study description	We analyzed long-term genetic and ecological/environmental data on a Yersinia pestis focus in north-west China, to trace the association between dynamics of the ecosystem and bacterial genome variation.
Research sample	We collected Y. pestis strains (n=78) that were sampled over 40 years from a plague focus named Guertu in north-west China. At least since 1967, Y. pestis strains could be isolated in this plague focus nearly every year.
Sampling strategy	We collected all current available Y. pestis strains that had been isolated from Guertu plague focus.
Data collection	Whole genome sequencing was done by Illumina HiSeq 2000. The precipitation and temperature information at the weather station nearest to Guertu (station number 51334) were obtained for the study period from China Meteorological Data Sharing Service System. The surveillance information (Supplementary Data 1) was collected from documents that were stored in Xinjiang CDC.
Timing and spatial scale	The Y. pestis strains were isolated from 1967-2006 in Guertu plague focus, in an area of <20km across. The climate information time series for this region spanned 1953-2012.
Data exclusions	No data was excluded from analysis.
Reproducibility	In determination of biofilm production of Y. pestis natural variants, the culture for each strain were separately added into six wells in polystyrene plate, correspondingly the measurement of OD620/OD570 for each cell was performed with six independent repeats. Multiple times of repeats shaped the foundation of statistical analysis and provided the confidence interval of the biofilm production for each strain (Fig. 1c).
Randomization	The only comparison between groups in the study is that of the rpoZ variants versus the rpoZ reference samples. The finding that rpoZ is relevant in any way for Y. pestis is new, and as such there no known covariates with rpoZ to control for. Furthermore, the ecosystem from which all samples were collected from is small (an area of < 20km across), and the rpoZ variants were observed in all branches in the phylogeny. We therefore had no known covariates to control for.
Blinding	All data was collected years prior to the sequencing and analysis in this paper, and as such data acquisition was not influenced by the hypothesis tested. For the statistical testing of which regions of the genome might be under selection pressure, there was no a priori expectation of which genes might be under selection pressure, and thus in effect was performed blinded.<br>For the determination of biofilm production of Y. pestis natural variants, the technicians who measured the biofilm production on the rpoZ variants and references did not know the hypothesis tested, therefore the experiment also in effect was performed blinded.<br>For the statistical analysis of the correlation between rpoZ variants and climate versus rpoZ reference and climate, the analysis was done with prior knowledge of the hypothesis, but the data was not mutable (climate, sampling times, rpoZ samples), and all possible directions of effect of temperature and precipitation on these samples were tested, and corrected for multiple testing.

Did the study involve field work? ☐ Yes ☒ No

Reporting for specific materials, systems and methods

We require information from authors about some types of materials, experimental systems and methods used in many studies. Here, indicate whether each material, system or method listed is relevant to your study. If you are not sure if a list item applies to your research, read the appropriate section before selecting a response.

Materials & experimental systems

n/a	Involved in the study	
☒	☐	Antibodies
☒	☐	Eukaryotic cell lines
☒	☐	Palaeontology
☒	☐	Animals and other organisms
☒	☐	Human research participants
☒	☐	Clinical data

Methods

n/a	Involved in the study	
☒	☐	ChIP-seq
☒	☐	Flow cytometry
☒	☐	MRI-based neuroimaging
