# Supplementary material for: Evolutionary selection of biofilm-mediated extended phenotypes in Yersinia pestis in response to a fluctuating environment
Source: Nat Commun. 2020 Jan 15;11:281. doi: 10.1038/s41467-019-14099-w (PMC6962365; doi:10.1038/s41467-019-14099-w)
Supplement: Supplementary file 4 — Description of Additional Supplementary Files [file 41467_2019_14099_MOESM4_ESM.pdf]

Description of Additional Supplementary Files

File Name: Supplementary Data 1
Description: Climate and surveillance information in Guertu nature plague focus

File Name: Supplementary Data 2
Description: Background of Guertu isolates and summary of sequencing data production

File Name: Supplementary Data 3
Description: SNP information of Guertu isolates

File Name: Supplementary Data 4
Description: Indel information of Guertu isolates

File Name: Supplementary Data 5
Description: Background information of the 368 public *Y. pestis* isolates

File Name: Supplementary Data 6
Description: Variations of *rpoZ* gene in *Y. pestis* strains
